# Ocular toxicity of intravitreal golimumab in a rabbit model

**DOI:** 10.3906/sag-1911-11

**Published:** 2020-06-23

**Authors:** Ceren DURMAZ ENGİN, Serap CİLAKER MIÇILI, Osman YILMAZ, Alper BAĞRIYANIK, Bekir Uğur ERGÜR, Fatoş ÖNEN, Ali Osman SAATCİ

**Affiliations:** 1 Department of Ophthalmology, Karadeniz Ereğli State Hospital, Zonguldak Turkey; 2 Department of Histology and Embryology, School of Medicine, Dokuz Eylül University, İzmir Turkey; 3 Department of Laboratory Animals Science, School of Medicine, Dokuz Eylül University, İzmir Turkey; 4 İzmir Biomedicine and Genome Center (iBG), İzmir Turkey; 5 Department of Internal Medicine, Division of Rheumatology, School of Medicine, Dokuz Eylül University, İzmir Turkey; 6 Department of Ophthalmology, School of Medicine, Dokuz Eylül University, İzmir Turkey

**Keywords:** Apoptosis, golimumab, intravitreal anti-TNF drugs, retina

## Abstract

**Background/aim:**

To investigate the effect of intravitreal golimumab on rabbit retina histopathology.

**Materials and methods:**

Sixteen albino New Zealand rabbits were divided into three groups. The right eye of each rabbit in groups I, II, and III received a single intravitreal injection of 5 mg/0.05 mL (6 eyes), 10 mg/0.1 mL (6 eyes), or 20 mg/0.2 mL (4 eyes) golimumab, while left eyes served as controls with the same volume of a balanced salt solution injection. All animals were examined using slit-lamp biomicroscopy and indirect ophthalmoscopy before and after intravitreal injection and at days 1 and 7. Animals were euthanized on day 7 and the eyes were enucleated for immunohistochemistry evaluation and electron microscopic examination of the retinas.

**Results:**

For groups I, II, and III, the number of cells in the outer nuclear layer and the inner nuclear layer was decreased compared to those in the control groups. In group I, the percentage of caspase-3 staining of the outer nuclear layer was significantly higher than that in the control. For groups II and III, TUNEL and caspase-3 staining percentages in the outer and inner nuclear layers were found to be significantly higher than those for the control groups. In the ganglion cell layer, for groups I, II, and III, neither TUNEL nor caspase-3 staining percentages showed any significant difference between two groups. No significant dose-dependent relationship was found for increasing doses of golimumab in all layers. Myelin figures and karyorrhexis in the photoreceptor cells were prominent in electron microscopy of the golimumab-injected eyes.

**Conclusion:**

Golimumab caused apoptosis in both photoreceptors and bipolar cells of the rabbit retina. Potential retinal toxicity of intravitreal golimumab should be considered if an intravitreal administration is planned.

## 1. Introduction

Tumor necrosis factor alpha (TNF-α) is a cytokine primarily released by macrophages, T lymphocytes, and fibroblasts. While it stimulates apoptotic cell death and inflammation, it also inhibits viral replication and tumor cell growth [1].

In diabetic retinopathy (DRP), increased TNF-α levels due to oxidative stress induce disorganization of retinal vascular structures and neovascularization [2]. Higher serum and aqueous TNF-α levels were found in noninfectious uveitis (NIU) cases compared to those in healthy controls [3,4]. TNF-α, by means of complement activation and induction of reactive oxygen species, plays a major role in the pathogenesis of age-related macular degeneration (AMD) [5]. 

TNF-α antagonists were initially used in the treatment of rheumatoid arthritis (RA) at the end of the 1990s [6]. Their therapeutic effect on ocular findings of RA, ankylosing spondylitis (AS), and psoriatic arthritis (PsA) have been noticed during their systemic administration [7–9]. However, since TNF-α antagonists increase the predisposition to infectious diseases and hypersensitivity reactions, their intravitreal (IVT) use in inflammatory diseases with only ocular involvement has been considered [10]. An IVT route is also preferable for using the vitreous as a drug reservoir and facilitating the drug access to ocular tissues [11,12]. Thus, etanercept, infliximab, and adalimumab have been used intravitreally in various ocular diseases, such as NIU, DRP, exudative AMD, and postoperative cystoid macular edema [13–16]. 

Golimumab is a novel human anti-TNF monoclonal antibody that can neutralize the human TNF-α molecule with high affinity and can be used in treatment regimens with longer intervals, due to its high chemical stability, longer half-life, and higher potency [17]. Currently, golimumab is approved for subcutaneous and intravenous route in moderate–severe RA, active PsA, and active AS [18]. It is effective against ocular manifestations of AS, juvenile idiopathic arthritis (JIA), and HLA B-27-positive arthritis when used systemically [19,20]. Following subcutaneous injection of golimumab to healthy subjects or patients the median time to reach maximum serum concentrations (Tmax) ranged from 2 to 6 days [18].

Safety and efficacy for IVT injections of etanercept, infliximab, and adalimumab have been studied in animal models and the treatment of human diseases [10,11,21–23]. To the best of our knowledge, IVT administration of golimumab has not yet been reported [24]. The aim of this study was to investigate the histopathological effects of IVT golimumab on the retinal layers of rabbit eyes, by using light microscopy, immunohistochemistry, and electron microscopy.

## 2. Materials and methods 

### 2.1. Animals 

Animals were treated according to the Association for Research in Vision and Ophthalmology Statement for the Use of Animals in Ophthalmic and Vision Research. The clinical and experimental protocol was approved by the Animal Care and Use Committee of Dokuz Eylül University (İzmir, Turkey). Sixteen normal albino New Zealand rabbits weighing 2–3 kg were divided into three groups. The original concentration of the drug was preserved in all groups but the amount of drug injected was changed by changing the volume. Group I (n = 6), group II (n = 6), and group III (n = 4) received 5 mg/0.05 mL, 10 mg/0.1 mL, and 20 mg/0.2 mL golimumab, respectively to the right eyes, and sham injections of a balanced salt solution (BSS) of the same volumes were administrated to the left eyes as controls. According to previous animal studies with anti-TNF drugs, if the original concentration was preserved (not diluted with saline or BSS), then 0.1 mL of these drugs would contain 2.5 mg etanercept, 1 mg infliximab, 5 or 10 mg adalimumab. Therefore, quantity of proportional doses in other anti-TNF studies are referenced while determining the injected doses [22,25,26]. 

### 2.2. Procedure 

Animals were anesthetized with a mixture of ketamine hydrochloride (50 mg/kg) and xylazine hydrochloride (5 mg/kg) along with topical anesthesia (Alcain; Alcon Laboratories, Inc., Fort Worth, TX, USA). The pupils were dilated with 2.5% phenylephrine hydrochloride (Mydfrin; Alcon Laboratories, Inc., Fort Worth, TX, USA) and 1% tropicamide (Tropamid; Bilim, İstanbul, Turkey). After instilling povidone iodine (5%), anterior chamber paracentesis of an equal volume to the injected drug was administrated to avoid drug reflux due to high intraocular pressure. IVT injection of golimumab (Simponi; *Merck Sharp* and *Dohme*, Kenilworth, New Jersey, USA) was performed approximately 2 mm posterior to the limbus with a 30-gauge needle attached to a tuberculin syringe. IOP was checked with TonoPen Avia (Reichert Technologies, Depew, NJ, USA) after the procedure and found within normal limits. A 0.3% ofloxacin eye drop (Exocin; Allergan, Dublin, Ireland) was administered topically immediately after the injection. Slit-lamp and funduscopic examinations were performed pre- and postinjection immediately and repeated at day 7. The rabbits were kept for 1 week in ambient light on a 12-h light/12-h dark schedule. 

### 2.3. Clinical observation

To detect clinical signs of toxicity, eyes were carefully evaluated by slit lamp examination at day 1 and day 7. Retinal artery patency was checked immediately after the injection with indirect ophthalmoscopy. At day 7, dilated fundus examination was repeated to see if there was any vitreous hemorrhage or retinal detachment.

### 2.4. Histological examination

At the first postinjection week, rabbits were euthanatized by intravenous injection of a lethal overdose of ketamin. The eyes were immediately enucleated and fixed in a 10% neutral formalin for 24–48 h. Five specimens for each eye were generated from random areas. For routine histological procedures, specimens were dehydrated with increasing alcohol series and then paraffin-perfused. Specimens were paraffin-embedded, and 5 mm sections were taken on poly-L-lysin-coated slides using a rotary microtome (RM 2135; Leica Instruments, Nussloch, Germany) with disposable metal microtome blades (type N35; Feather Company, Osaka, Japan). Then tissues stained with hematoxylin-eosin (H&E). Samples were examined under a light microscope (Olympus BH2, Japan) and pictures which showed focal retinal changes of relevant tissue section were transferred to the computer using a digital video camera (Olympus DP71, Japan).

#### 2.4.1. Immunohistochemical assessment

After deparaffinization, tissues were dewaxed in xylene and rehydrated with ethanol. To unmask antigens by the heat treatment, sections were treated with 10 mM citrate buffer (Cat No. AP-9003-125 Labvision). After incubated in a solution of 3% H2O2 to inhibit endogenous peroxidase activity, sections were incubated with normal serum blocking solution. They were then incubated in a humid chamber for 18 h at +4 °C with antibody active-caspase-3(Novus), biotinylated IgG, and streptavidin conjugated to horseradish peroxidase (Invitrogen-Plus Broad Spectrum 85-9043) in order. Sections were finally incubated with 3,30 diaminobenzidine hydrochloride (DAB) (1718096, Roche), and nuclei were counterstained with Mayer’s hematoxylin. Sections were dehydrated through a graded ethanol series, cleared in xylene, mounted in entellan (Merck, Germany) and analyzed by a light microscope. Immunohistochemical evaluation was performed by two independent experienced histologists, blinded to the source of the samples taken from five random fields of the same sections, and the average score was utilized.

#### 2.4.2. Detection of the apoptotic cell death in situ using the TUNEL method

Apoptosis was investigated by using in situ TUNEL (TdT-mediated dUTP-digoxigenin Nick End Labeling) DeadEnd Colorimetric TUNEL system kit (Cat. No:11 684 817 910 Roche, Germany). Tissues were deparaffinized, rehydrated in alcohol, and microwave-pretreated in proteinase-K solution at 37 °C for 10 min (Roche, 10 109 819 001). Specimens were washed in phosphate-buffered saline (PBS), incubated with fluorescein labeled deoxy-UTP and TdT at 37 °C for 60 min and then treated with converter peroxidase (POD) solution. They were stained with DAB, counter-stained with hematoxylin and mounted in entellan. The percentage of TUNEL-positive cells was determined by counting dye-positive cells from five random fields. 

#### 2.4.3. Electron microscopic examination

Tissue samples were fixed in 2.5% glutaraldehyde. Specimens were washed and submerged in a solution containing equal amounts of 2% osmium tetra oxide and phosphate. Samples were then passed through series of graded alcohol solutions and afterwards left in propylene oxide. They were embedded in Araldite-CY 212 and dodecanyl succinate anhydride (DDSA) for 1 night. The next day they were placed in gelatin capsules that had been filled with a combination of Araldite-CY 212, DDSA, and benzyl dimethylamine (BDMD), and these capsules were incubated in an autoclave, first for 24 h at 40 °C and then for 48 h at 60 °C. The specimen lumps were cut into semithin and ultrathin sections with a Leica EM UC. The semithin sections of 0.5 mm were then stained with toluidine blue and examined under Olympus BH-2 (Tokyo, Japan) light microscope. Ultrathin sections of 120 nm were obtained from selected blocks mounted on copper grids, stained with uranyl acetate and lead citrate and examined using a Sigma 300 scanning transmission electron microscopy (STEM) and images were digitally photographed. 

### 2.5. Statistical analysis

All statistical procedures were performed with SPSS software for Windows (version 21.0 SPSS, Chicago, IL, USA). All values were reported as the mean (standard deviation) or median (range). To compare cell counts at different retinal layers in H&E staining between golimumab and BSS groups, Student’s *t*-test was used. Immunohistochemical staining differences between golimumab and BSS groups was compared with Student’s *t*-test in normal-distribution groups and Mann–Whitney U test for nonnormal distribution groups. Statistical significance was set at 0.05. For comparison of H&E, TUNEL, and caspase staining percentages of the cells in different retinal layers in increasing doses and/or volumes, the Kruskal–Wallis test was used. If a statistically significant difference was found, paired groups were compared using the Mann–Whitney U test to find the group that created the difference. Significance values have been adjusted using the Bonferroni correction for pairwise comparison tests.

## 3. Results

None of the rabbits died during the 1-week study period. No inflammation or ocular media opacity was observed with slit lamp microscopy. Retina and optic nerve appeared normal. No optic atrophy, vitreous hemorrhage, or retinal detachment was seen in any of the eyes.

### 3.1. Histopathologic findings

#### 3.1.1. Light microscopy

In H&E staining, decreased cell count was found in both the outer nuclear layer (ONL) and inner nuclear layer (INL) in the golimumab-injected eyes compared to the BSS injected eyes for three different doses. There was no statistically significant difference in the ganglion cell layer (GCL). Cell counts at different retinal layers in H&E staining are shown in Table 1.

**Table 1:** Cell counts at different retinal layers in H&E staining.

Light Microscopy	Golimumab	BSS	P-value
	Mean ± standarddeviation (%)	Mean ± standarddeviation (%)	
	5 mg/0.05 mL	0.05 mL	
Outer nuclear layer	382.50 ± 45.57	519.16 ± 52.13	0.001*
Inner nuclear layer	107.33 ± 33.33	142.5 ± 33.92	0.047*
Ganglion cell layer	9.5 ± 2.66	12.1 ± 3.61	0.11
			
	10 mg/0.1 mL	0.1 mL	
Outer nuclear layer	410.00 ± 54.40	510.00 ± 65.11	0.044*
Inner nuclear layer	99.33 ± 16.30	111.66 ± 25.34	0.045*
Ganglion cell layer	8.16 ± 3.92	9.16 ± 3.97	0.067
			
	20 mg/0.2 mL	0.2 mL	
Outer nuclear layer	335.00 ± 66.08	402.50 ± 73.65	0.045*
Inner nuclear layer	78.00 ± 14.35	88.00 ± 12.98	0.048*
Ganglion cell layer	6.50 ± 1.29	7.00 ± 1.63	0.2

Comparison between Golimumab and BSS groups was made with Student’s t-test. (P ≤ 0.05)

There was no significant difference between any of the study and control groups in terms of ONL, INL, and GCL cell counts in increasing doses and/or volume (P = 0.054, P = 0.062, and P = 0.370 for the study groups and P = 0.068, P = 0.072, and P = 0.490 for the control groups respectively). 

#### 3.1.2. Immunohistochemical findings

##### 3.1.2.1. TUNEL staining

The TUNEL staining technique—demonstrating DNA fragmentation in the apoptotic process—showed that the TUNEL-positive cell percentage in the INL was significantly higher in groups I, II, and III compared to controls. Although the percentage of TUNEL-positive cells in the ONL was not different from the control in group I, increased TUNEL positivity in groups II and III was observed compared to controls. The percentage of TUNEL-positive cells in the GCL of all study groups was not significantly different from the control eyes. TUNEL staining percentages of the cells in different retinal layers detected in the study are summarized in Table 2.

**Table 2:** Percentage of TUNEL-positive and caspase-3–positive cells at three different layers of study and control groups.

Immunohistochemistry	Golimumab	BSS	P-value
TUNEL			
	5 mg/0.05 mL (n=6)	0.05 mL (n=6)	
Outer nuclear layer	72.18 ± 1.66	68.77 ± 1.71	0.061
Inner nuclear layer	72.92 ± 1.39	55.72 ± 3.66	0.018*
Ganglion cell layer	100 (75–100)	100 (66–100)	0.937
	10 mg/0.1 mL (n=6)	0.1 mL (n=6)	
Outer nuclear layer	85.50 (72–88)	69.79 (57–78)	0.009*
Inner nuclear layer	75.62 ± 1.84	66.09 ± 2.91	0.020*
Ganglion cell layer	100 (66–100)	100 (60–100)	1
	20 mg/0.2 mL (n=4)	0.2 mL (n=4)	
Outer nuclear layer	87.22 (83–88)	72.77 (69–73)	0.029*
Inner nuclear layer	73.77 (63–77)	60.87 (47–66)	0.029*
Ganglion cell layer	100 (66–100)	100 (60–100)	1
	5 mg/0.05 mL (n=6)	0.05 mL (n=6)	
Outer nuclear layer	56.58 ± 1.54	48.00 (13–61)	0.048*
Inner nuclear layer	60.15 ± 4.44	42.36 (11–54)	0.015*
Ganglion cell layer	76.66 ± 8.33	63.54 (0–50)	0.064
	10 mg/0.1 mL (n=6)	0.1 mL (n=6)	
Outer nuclear layer	54.64 ± 3.16	44.27 (41–49)	0.041*
Inner nuclear layer	59.51 ± 3.55	48.77 ± 2.90	0.041*
Ganglion cell layer	58.33 ± 20.00	23.33 ±10.54	0.163
	20 mg/0.2 mL (n=4)	0.2 mL (n=4)	
Outer nuclear layer	52.89 (49–61)	38.49 (22–54)	0.048*
Inner nuclear layer	50.28 (44–52)	33.92 (28–55)	0.041*
Ganglion cell layer	66.66 (0–100)	25 (0–50)	0.198

All numerical values are expressed as Mean ± (Standard Deviation) for groups with normal distribution, and Median (lowest–highest) for groups with nonnormal distribution. To compare golimumab and BSS groups, Student’s t-test was used for groups with normal distribution and Mann–Whitney U test was used for nonnormal distribution groups. (P ≤ 0.05)

In the increasing doses and/or volumes of golimumab, the percentage of TUNEL-positive cells was significantly different in the ONL (P = 0.014), while there was no significant difference in the INL and GCL (P = 0.337 and P = 0.490, respectively). To investigate which groups created the difference, the Mann–Whitney U test was executed. There was a significant difference in the percentage of TUNEL positive cells only between group I and group III (P = 0.020); however, there was no significant difference between group I and group II (P = 0.087); and group II and group III (P = 1.00). 

To analyze the volume effect on retinal layers, increasing BSS injection volumes (0.05 mL, 0.1 mL, and 0.2 mL) were performed. There was no significant difference in the ONL, INL, and GCL staining percentages (P = 0.063, P = 0.106, and P = 0.491, respectively). This was evidence of apoptosis being independent of injected volume; thus, the chemical effect of golimumab was the only reason for apoptosis in this study.

**Figure 1:**
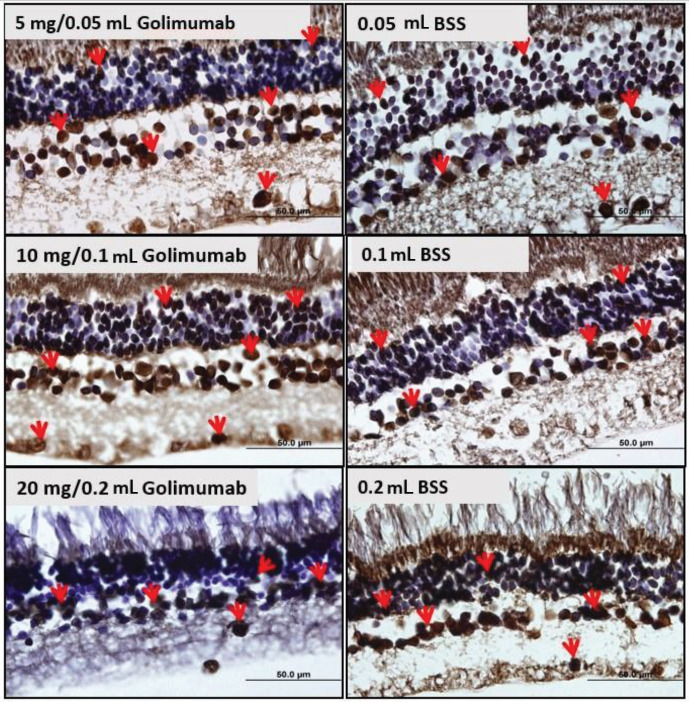
Examples from the study and control eyes of each group for TUNEL immunostaining. TUNEL-positive cells were identified with red arrows.

TUNEL staining photographs of the rabbits’ study and control eyes are shown in Figure 1.

##### 3.1.2.2. Caspase-3 staining

Since caspase-3 activation is the most important indicator of the irreversible point in programmed cell death, cytoplasmic caspase expression was shown by using antibodies developed against it. Caspase-3 staining was observed in the cytoplasm of photoreceptor cells (PR), bipolar cells, and ganglion cells.

In the immunohistochemical examination, while caspase-3 staining in the ONL and INL was significantly higher in all three golimumab injected groups compared to the BSS groups, there was no statistically significant difference in the GCL. Caspase-3 staining percentages of the cells in different retinal layers are shown in Table 2.

**Figure 2:**
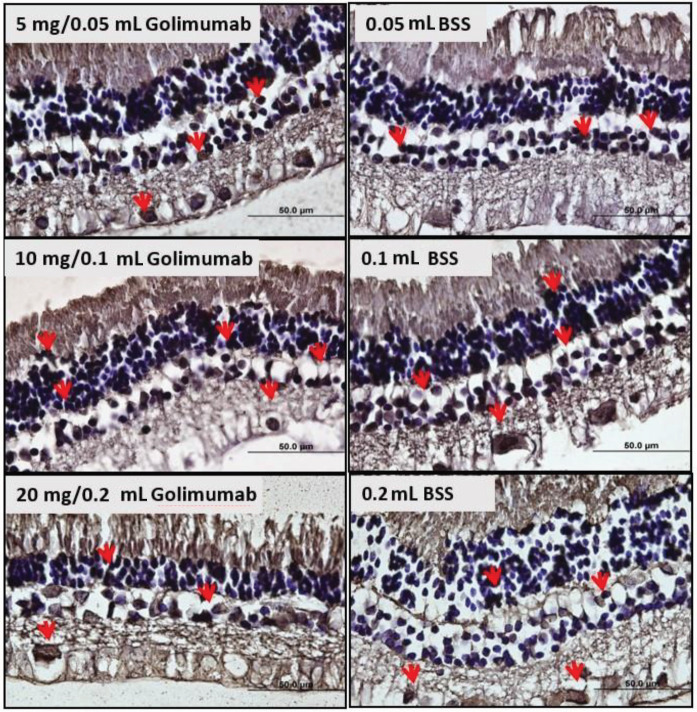
Examples from the study and control eyes of each group for caspase-3 immunostaining. Caspase-3–positive cells were identified with red arrows.

In the increasing doses and/or volumes of golimumab, the percentage of caspase-positive cells was not significantly different between the three retinal layers (P = 0.555, P = 0.092, and P = 0.877, respectively). As for the volume effect on apoptosis, there was no significant difference in caspase positive cell percentages of the ONL, INL, and GCL at increasing BSS volumes (P = 0.155, P = 0.236, and P = 0.955, respectively). Caspase-3 staining photographs of the rabbits’ study and control eyes are demonstrated in Figure 2.

### 3.2. Electron microscopy

**Figure 3:**
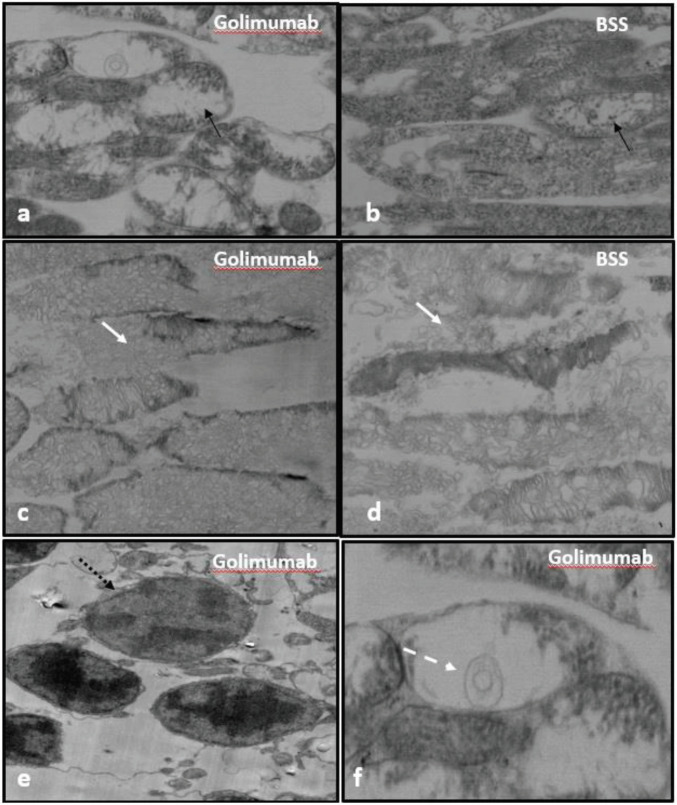
a-b. Mitochondrial crystalysis (black arrow) in the photoreceptor inner segment. TEM, Golimumab X31.300, BSS X26.230 magnification c-d. Disruption of outer segments of photoreceptors (white arrow) TEM, Golimumab X17.130, BSS X24.140 magnification e.Photoreceptor cell nucleus, more translucent than surrounding cells, and karyorrhexis (black dashed arrow) f. Myelin figure (white dashed arrow) in the photoreceptor inner segment.

Mitochondrial crystalysis in the internal segment (Figures 3a and 3b) and destruction of disc structures of the external segment (Figures 3c and 3d) of photoreceptors were observed in both the drug and control groups. The most prominent ultrastructural change observed in the retina of the golimumab group was karyorrhexis in the nucleus (Figure 3e) and myelin figures in the inner segments (Figure 3f) of the photoreceptor cells. Other layers of the retina were evaluated as normal in all study groups. Figure 3 depicts EM photos of the rabbits’ study and control eyes.

## 4. Discussion

In the literature, several studies have evaluated the retinal toxicity of anti-TNF drugs which were used off-label [22,25–30]. Immunohistochemical analysis was performed in only two studies to investigate the amount of drug presence in the retina. Our study, unlike others, evaluated apoptosis immunohistochemically and investigated both quantitative and qualitative information about retinal layers with light microscopic examination. Functional evaluation with electroretinography (ERG) could not be performed due to the lack of equipment. The aforementioned studies are summarized in Table 3.

**Table 3:** Studies evaluating the retinal toxicity of anti-TNF drugs in the literature.

Study	Drug	Type ofAnimal	Number of animal/eyes	Dose	Light microscopy	Immunohistochemistry	ERG	Electron Microscopy
Our study	Golimumab	New Zealand albino rabbit	16	5 mg / 0.05 mL, 10 mg / 0.1 mL or 20 mg/0.2 mL	Decreased cell count in ONL and INL; no difference in GCL	Higher percentage of TUNEL and Caspase staining in 10 and20 mg groups	N/A	Myelin figures in the inner segments and karyorrhexis in the nucleus of the photoreceptor cells
Melo et al. (2012) 29	Infliximab	Marmosets	10	100 or 400 mcg in 5 mcl	No retinal abnormality in drug and control groups	Presence of drug with IHC	Normal -clinically no relevant- in drug and control groups	No retinal abnormality in drug and control groups
Manzano et al. (2011) 30	Adalimumab	New Zealand albino rabbit	30	0.5, 1.0, 2.5, 5and 10 mg in0.1 mL	No retinal abnormality in drug and control groups	N/A	Normal up to 5 mg, decrease in the photopic-wave in the 10 mg group	Normal up to 5 mg, inconclusive in10 mg due totechnical failure
Theodossiadis et al. (2009) 28	Infliximab	New Zealand albino rabbit	28	1, 2, 5, 8, 10 or20 mg in 0.1 mL	Normal in saline, 1 and 2 mg; edema of ganglion cells, nerve fibers layer and the inner plexiform layer in higher doses	N/A	Normal in saline, 1and 2 mg; dose-dependent reductions in b-wave amplitudein higher doses	N/A
Manzano et al. (2008) 26	Adalimumab	New Zealand albino rabbit	12	0.25, 0.50 or1 mg in 0.1 mL	Normal in BSS, 0.25 and 0.50 mg of adalimumab; inflammatory cell infiltration in two of three eyes in 1.0 mg group	N/A	Normal in BSS, 0.25 and 0.50 mg; greater than 30% reduction in a and b wave in 1 mg	N/A
Giansanti et al. (2008) 25	Infliximab	New Zealand albino rabbit	12	L1, 1.7 and 3.3 mg in 0.1 mL	No retinal abnormality in 1 mg and 1.7 mg; significant edema of the nerve fibers in two of three eyes injected with 3.3 mg infliximab	N/A	No difference compared to control	N/A
Kivilcim et al. (2007) 22	Etanercept	New Zealand albino rabbit	20	125 mcg, 250 mcg, 500 mcg,1 mg and 2.5 mg in 0.1 mL	No retinal abnormality in drug and control groups	N/A	Normal	N/A
Fauser et al. (2004) 27	Etanercept	Pigmented rabbits	Notmentioned	100 mcg/0.1 mL	No retinal abnormality in drug and control groups	Presence of drug with IHC	Normal	N/A

In our study, golimumab-injected eyes showed a decreased number of cells in the ONL and INL in H&E staining. To test if the decrease in cell number was due to apoptotic cell death, we performed TUNEL and caspase-3 staining methods together to increase sensitivity and specificity.

The TUNEL method, which is the standard technique for demonstrating apoptosis, shows 3’-hydroxy groups, i.e. DNA degradation products [31]. Necrosis, autolysis and DNA chain fractures to be repaired later can also be stained as TUNEL positive [32]. In contrast, if larger destruction fragments are formed instead of nucleosomal fragments by apoptosis, the number of apoptotic cells detected by TUNEL will be accidentally low [33]

Caspase-3 staining is a cytoplasmic apoptosis marker and is highly specific. It functions in the early stages of apoptosis, whereas DNA degradation products stained with TUNEL are late findings of this process. [34]. However, apoptosis may occur without caspase activation or by other caspases other than caspase-3, which reduces the sensitivity of caspase-3 staining [35]. In accordance with the literature, the percentage of TUNEL staining in our study was higher than the percentage of caspase-3 staining in all groups [36].

In all study groups, the percentage of cells stained positive in the INL was significantly higher than the BSS group in both staining methods. While the percentage of TUNEL positive cells in the ONL was not different from the control in group I, increased TUNEL positivity was observed in groups II and III compared to controls. In caspase-3 staining, the percentage of positive-stained cells in all three doses was significantly higher than in the control group. We think that the difference in the ONL is because the cells in this layer might still be in early stages of apoptosis, so they were stained with caspase-3 but not with TUNEL.

Several studies in the literature have evaluated the apoptotic effect of anti-TNF drugs by immunohistochemistry. Paula et al. compared the apoptotic effect of IVT adalimumab, sham injection, and no injection to the rabbits’ eyes and found that the caspase gene expression was similar between three groups [37]. Ueda et al. found that infliximab and adalimumab increased the amount of active caspase-3 compared to golimumab, thus leading to more apoptotic DNA fragmentation [38]

There was no significant difference in the percentage of TUNEL and caspase-3 positive cells compared to the control group in the golimumab group’s GCL. There are a limited number of studies that investigate drug toxicity on ganglion cells. In the in vitro rat ganglion cell culture model, Tezel et al. found that a caspase-independent pathway in ganglion cells was speculated [39]. In the experimental retinal degeneration model, Martínez-Fernández et al. suggested that TNF-α induces cell death by the caspase-independent pathway in the ONL and GCL and the caspase-dependent pathway in the INL [40]. 

In our study, the difference in the percentage of caspase-3 positive cells in the GCL compared to the control group might suggest that caspase-independent mechanisms are effective in the process. Contrary to our findings, however, TUNEL staining percentages should be different between the two groups in this case. Although the ganglion cell count in the study group decreased compared to the control group, the difference was not significant. This may be explained by the neuroprotective effect of TNF-α antagonism shown in the studies mentioned, but also may be because of ganglion cell counts being less than the cells in the other layers analyzed (INL and ONL) in normal retina.

In the increasing doses of golimumab, the only significant difference in apoptosis was found in 20 mg/0.2 mL group compared to 5 mg/0.05 mL group with TUNEL staining. Otherwise, no significant decrease in cell count or increase in the apoptotic cell percentage with increasing golimumab dose in H&E, TUNEL, and caspase-3 staining were seen. This suggests that the toxicity of IVT golimumab may not be dose-dependent for 5 mg, 10 mg, and 20 mg. However, we believe that this finding should be supported by further studies with larger numbers of groups where the drug is injected in a larger dose range.

In this study, various retinal cells of rabbit eyes injected with golimumab and BSS were evaluated with TEM. While myelin figures and karyorrhexis—a sign of cell damage—were observed in the drug group, increased intracellular space and decreased cell numbers in the INL and ONL were also prominent. Melo et al. observed that all retinal layers are normal in marmoset eyes after IVT 100 mg and 400 mg infliximab or BSS injections [29]. Inan et al. found mitochondrial swelling and disruption in the crystalline structure of PR internal segments in bevacizumab injected eyes [41].

The excipients of our injected drug include sorbitol, histidine, histidine hydrochloride monohydrate, polysorbate 80, and water for injections. Histidine and histidine hydrochloride monohydrate are already tested for IVT route and approved for human diseases as an anti-VEGF drug [42]. Damico et al. showed that IVT injection of polysorbate 80 in the concentration for IVT drug preparations did not cause any functional alterations of rabbit retinas [43]. 

In human eye, vitreous humor is approximately 4 mL, while volume of the rabbit vitreous is approximately 1.5 mL. In rabbits, the retina is less vascular and the lens is larger. These factors may cause the pharmacokinetics of drugs to be different from human eyes. Comparative studies in the literature suggest that rabbit eyes are useful and effective animal models for studying IVT pharmacokinetics (clearance, distribution volume, and half-life), but since the anti-TNF drug we administered is targeting human TNF-α, its affinity to rabbit TNF-α may vary [44–47]. Although this situation is not seen as a significant confounding factor in our study evaluating drug toxicity, it should be taken into consideration in future studies.

Relatively short study duration, limited administered dose range and the lack of functional tests (ERG, optical coherence tomography, etc.) are the main limitations of the present study.

To conclude, in this study, we observed that golimumab caused apoptosis in both photoreceptors and bipolar cells of the rabbit retina. Our study is first to evaluate the retinal toxicity of a novel anti-TNF drug, golimumab, in an animal model by light microscopy, immunohistochemistry, and electron microscopy. Thus, it pioneers future studies of its IVT use in human diseases.

Considering that each intravitreal injection carries the risk of ocular infection namely endophthalmitis; it is advantageous that golimumab, which has a longer half-life and a higher potency, can be used at longer intervals in inflammatory diseases of the eye.

## Conflict of interest

None of the authors has any financial interest in any of the products mentioned in this paper.
